# Sexual and reproductive health literacy among young people in Sub-Saharan Africa: evidence synthesis and implications

**DOI:** 10.1080/16549716.2023.2279841

**Published:** 2023-11-27

**Authors:** Adamu Amanu, Zewdie Birhanu, Ameyu Godesso

**Affiliations:** a Department of Health, Behaviour, and Society, Faculty of Public Health, Jimma University, Jimma, Ethiopia; b Department of Sociology, College of Social Sciences, Jimma University, Jimma, Ethiopia

**Keywords:** Sexual and reproductive health literacy, scoping review, Sub-Saharan Africa, young people, sexual health literacy, reproductive health literacy

## Abstract

**Background:**

Sexual and reproductive health literacy is a key to attaining and maintaining sexual and reproductive health, especially among young people in low-income countries, such as sub-Saharan Africa. While the importance of sexual and reproductive health literacy is gaining wider recognition, studies on the topic have been mainly concentrated in high-income (developed) countries.

**Objective:**

The aim of this study was to provide a coherent summary and synthesis of the available evidence on sexual and reproductive health literacy among young people in sub-Saharan Africa, with implications for policy, interventions, and research.

**Methods:**

The review was conducted using the Joanna Briggs Institute’s methodology for reviews and the Preferred Reporting Items for Systematic Reviews and Meta-Analyses reporting guideline to enhance the clarity and transparency of the reporting process. PubMed, CINAHL, AJOL, AIM, and Google Scholar were searched for evidence from 18 March to 20 May 2022.

**Results:**

The search provided 2,682 articles in total, of which only 24 met the eligibility criteria and were included in this review. The findings revealed persistent sexual and reproductive health information/knowledge gaps, poor sexual and reproductive health-related knowledge and practices, lack of exercising sexual and reproductive health knowledge, and multiple determinants of sexual and reproductive health literacy among young people, ranging from personal to larger structural conditions.

**Conclusion:**

The review found that sexual and reproductive health literacy among young people in sub-Saharan Africa is concerning and has not been fully researched. A deeper understanding of the issue is essential for designing and implementing effective interventions to improve sexual and reproductive health literacy and health outcomes among young people.

## Background

Young people are defined as those between the ages of 10 and 24 years, representing a significant global demographic [1]. Sub-Saharan Africa (SSA) has the highest concentration of young people in the world, a vast human resource with decisive implications for the region’s socio-economic development now and in the future [2,3]. Young people are often vulnerable to a cluster of risky behaviours that are associated to the profound changes they experience in different life aspects [4–10]. Accordingly, young people, mainly those in low-income countries, face tremendous challenges, especially in relation to sexual and reproductive health (SRH) [11]. For example, about 11% of all pregnancies worldwide occur in young people, specifically adolescents, and about 95% of these pregnancies occur in low- and lower-middle-income countries [12]. Adolescent pregnancy is often followed by life-threatening abortion and adverse health outcomes, including sexually transmitted infections/diseases (STIs/STDs) and other related problems. It is the leading cause of death among young people in SSA, where 85% of all HIV-infected adolescents worldwide live [11,13,14]. These problems trap young people in a vicious circle of poverty and make them more vulnerable to unsafe behaviours [15].

Global commitments to and support for SRH among young people have increased in recent years [16]. However, despite significant progress in many areas, an estimated 1.75 million adolescents worldwide were HIV positive in 2020, accounting for 11% of new HIV infections [17]. Similarly, while SSA has made progress in improving SRH outcomes for adolescents, these gains have been small and uneven across the region [2,18], and young people in SSA remain the population at the greatest risk of STDs [19].

At the 9th Global Conference (Shanghai Declaration), the World Health Organization recognised health literacy (HL) as a key action area to reduce health inequalities [20]. HL refers to a broad range of knowledge, competencies, and motivation to access, understand, appraise, and apply health-related information to make judgements and decisions in everyday life regarding healthcare, disease prevention, and health promotion [21]. It includes knowledge of the healthcare system and health risks, the confidence to act independently on the knowledge, and the ability to effectively act for the benefit of oneself and the community [22]. Sexual and reproductive health literacy (SRHL) therefore goes beyond knowledge to include the motivation and competencies to access, understand, appraise, and apply SRH-related information to cope with SRH problems [23,24].

HL is a key to prevent health-compromising behaviours and promote healthy development among young people [25–27]. It empowers young people to seek, engage with, and use valid health information, or to make informed health decisions, and to work on and improve the factors that constitute their health chances [28,29]. Therefore, HL is a decisive issue that can help combat or mitigate SRH-related challenges among young people, especially in low-income countries, such as SSA, where there have been critical SRH problems like teenage pregnancy, life-threatening abortion, and STDs among adolescents [14,15,30–32].

Although the importance of HL, including SRHL, is increasingly being recognised worldwide, HL research has been mainly concentrated in developed (high-income) countries [11,21,33], and HL assessments have been mostly focused on adults within medical contexts [34,35]. There has been a paucity of HL research in Africa [36] and, to the best of the authors’ knowledge, a lack of reviews of available HL studies in general and SRHL studies in particular among young people in SSA. However, a synthesis and understanding of the available evidence is essential to identify what is known and unknown or to understand the progress made so far and inform the gaps. Therefore, the aim of this review was to provide a coherent summary and synthesis of available literature on the issue and to indicate implications for policy, interventions, and research in SSA.

## Methods

The review was conducted in accordance with the Joanna Briggs Institute’s (JBI) systematic scoping reviews methodology [37]. The review used the Preferred Reporting Items for Systematic Reviews and Meta-Analyses (PRISMA) guideline to enhance the clarity and transparency of the reviews reporting process [38].

## Inclusion and exclusion criteria

Inclusion criteria included studies conducted in SSA, studies concerning young people (within the age range of 10–24), studies published in English, studies reported on at least two aspects of SRHL (accessing, understanding, appraising, and using SRH information, including SRH knowledge and practices/behaviours), and original studies published in full manuscript. Exclusion criteria included studies concerned with only SRH knowledge or with only one aspect/element of SRHL, and studies that fail to meet any of the above eligibility criteria. Based on the purpose of this review, to be as inclusive as possible, any study that fulfilled the above inclusion criteria was eligible, regardless of time of publication, quality, and design of the study.

## Data sources and search

The databases/sources searched for evidences were PubMed, CINAHL, AJOL (African Journals Online), and AIM (African Index Medicus). The Google Scholar website was also searched for additional relevant articles. For each of the databases identified, different search strategies and terms were developed and used. For instance, in PubMed, the following terms were used: ‘health literacy’, ‘reproductive health literacy’, ‘sexual health literacy’, sexual, reproductive, ‘sexual health’, ‘reproductive health’, adolescent, ‘young adult’, ‘young people’, teen, student, youth, ‘Sub-Saharan Africa’, and names of all of the sub-Saharan African countries. Mesh terms, Boolean operators (AND, OR), field codes ([tw], [tiab]), and truncation (*) were also used wherever needed. The references of the eligible studies were also hand-searched for additional relevant studies. The searches were conducted from 18 March to 20 May 2022. Details of the full search strategies and search dates for all the searched databases are given in Supplementary Material.

## Study selection and data extraction, presentation, and analysis

**Figure 1.**
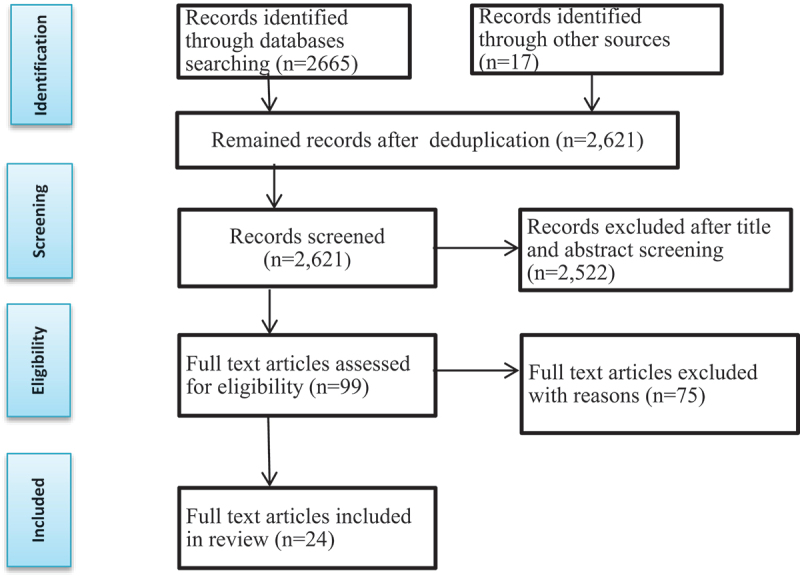
The PRISMA flow diagram [38].

Using EndNote X9 software program, duplicate studies were identified and removed from the search results. Following deduplication, the study screening and selection process was carried out using the PRISMA flow diagram guideline, which helped to ensure a transparent reporting of the review process [38]. Each deduplicated study’s title and abstract were screened for relevance, and irrelevant studies were removed and the remaining potentially eligible studies were reviewed in full based on the eligibility criteria. Finally, for the final review, articles that met the inclusion criteria were selected. Figure 1 shows the study selection processes and results, in further detail.

Data extraction table was adapted from the standardised data extraction tool from the JBI’s methodology guidance for scoping reviews and used for this study [37] (see Supplementary Material). From the eligible studies, the extracted information include name of the author(s), year of publication, country of study, research objective, study design/method, data collection instruments, study setting, study population, sampling technique, and main findings. The extracted data from the eligible studies were summarised and presented in a table format (Table 1), and the table is accompanied by narrative synthesis of findings [37]. Findings were presented and analysed by bringing together related issues.

**Table 1.** Overview of the eligible studies.

Author/s & Year of publication	Country	Study objective	Study design & method	Study setting; study population & sample size in analysis & sampling technique	Instrument utilised	Main findings
Barchi, Ntshebe [39]; 2022	Botswana	To examine school adolescents contraceptive literacy and condom knowledge	Cross-sectional; quantitative	School in urban area; 233 secondary school students aged 14–19 years; all a club participants	Questionnaire adapted from WHO’s illustrative questionnaire and the 2005 Botswana global school-based health survey	Ninety per cent of the adolescents were aware of one or more forms of contraception; however, only 67% of them could name a method they knew its source and only about half of the sexually active ones had used birth control during their latest sexual experience. Teachers and family members were mentioned as their most important information sources, and only about 8.2% of the adolescents identified health professionals as information source, however, adolescents who consulted nurses had 8 times greater odds of reporting right condom using knowledge than the ones who consulted teachers.
Kibret [40]; 2003	Ethiopia	To assess high school students’ reproductive health knowledge, attitude and practice	Cross-sectional; mixed methods	School; 200 high school students aged 14–20 years; randomly selected	Pretested questionnaire and GD guide	Although the students had high knowledge of contraceptives and where to obtain the services, their utilisation level was low, because of different factors including lack of access to services, carelessness, unplanned sexual intercourse, and pressure from their sexual partners. They engaged in sexual relationships at an early age without protection or with unsafe methods. Their educational level was the demographic variable that significantly associated with their sexual experience.
Molla, Emmelin [41]; 2009	Ethiopia	To examine knowledge regarding common STIs, and the perceptions, preferences and use of health services for the STIs among youths and healthcare providers	Cross-sectional; mixed-method	Community-based in rural area; 3743 youths aged 15–24 years; randomly selected	Pre-tested questionnaire and interview guide	Only less than 38% knew the common STIs. From the sexually active ones, 3.9% reported having at least one symptom of STI in the past 12 months, and one-half of those who had had STI symptom reported not seeking care from any source. The youths reported preferring to consult healthcare providers of the same sex, who were young, friendly and empathetic.
Kassa, Luck [42]; 2016	Ethiopia	To assess the knowledge, attitudes, and practice of sexual and reproductive health related issues among young people with disabilities (YPWD)	Cross-sectional; quantitative	Disability associations; 426 YPWD, members of disability associations, aged 10–24 years; systematic random sampling	Questionnaire	Only 64.6% were aware of SRH services; 62.2% reported radio and TV as their main sources of information. 77.9% reported never having a discussion about SRH issues with their parents. Although 96.7% reported hearing about HIV, 88% had poor knowledge of preventing it. Among them, perception of the risk of getting infected with HIV was low.
Yared, Sahile [43]; 2017	Ethiopia	To assess university students’ SRH experiences, knowledge, and problems	Cross-sectional; mixed approach	University; 410 university students; mean age was 21.14 ± SD 1.931; stratified sampling; simple random sampling	Questionnaire; interview guide	Almost all ever had heard of STIs (94.5%) and HIV/AIDS (98%), and 89.4% knew some modern contraceptives. In spite of awareness of STIs, > 22.8% had STIs in the past year prior to the study. In the quantitative study, low unwanted pregnancy (5%) and abortion (2.5%) reported to be existed in the campus, however, in the qualitative part, high rates of unwanted pregnancy and unsafe abortion were reported.
Rondini and Krugu [44]; 2009	Ghana	To examine the adolescent students’ knowledge, attitude and practices of reproductive health	Cross-sectional; mixed meth.	School; 250 secondary school students aged 14–24.years; not clearly specified	Questionnaire, FGD guide	The students had low familiarity with family planning methods and HIV/AIDS transmission, and had low contraceptive use, and were at high risk for unplanned pregnancies and sexual infections/diseases.
Van der Geugten, De Vries [45]; 2015	Ghana	To assess the students’ knowledge, attitudes, and behavioural intentions regarding SRH and the effects of SRH programme on the students	A quasi-experimental study used a pre-post-intervention design	School; the junior and senior high schools students and vocational schools students; 312 before, and 272 after; with age range of 10–23 years; not clearly specified	Questionnaire	Before the SRH programme, they answered correctly half of the knowledge questions, and their thought concerning deciding whether to have a relationship and to have sex, and their intentions of SRH behaviour, like condom use were positive. The SRH programme/intervention led to a small but significant increase in their knowledge. The attitude of those aged 18–20 significantly improved, and female students aged 18–20 were more positive towards changing their behaviour after the intervention.
Ejembi and Otu [46]; 2004	Nigeria	To examine undergraduate students’ reproductive health knowledge, sexual behaviour, contraceptive use, and reproductive outcomes	Cross-sectional; quantitative	University; 387 undergraduate students aged 15–24 years; multistage sampling/availability sampling	Pre-tested, questionnaire	The participants had high knowledge level for most aspects of reproductive health, despite some gaps in some areas. However, about 64.1% of the students had had sexual intercourse, and only 32.4% of them had ever used or were currently using a method of contraception. Within six months preceding the study, 23.3% had experienced STIs symptoms, and self-medication was reported as the dominant method of treatment.
Lawal and Olawale [47]; 2017	Nigeria	To assess the extent to which preference for and access to media resources predicts knowledge of adolescents regarding sexual and reproductive health matters	Cross-sectional; quantitative	School; 510 senior secondary school adolescents; age range not clearly specified; simple random sampling	Questionnaire	The adolescents reported preferring print-based information resources over other media resource forms. Although the school library was identified as the major place of accessing SRH information, there was a low degree of accessibility to media resources on SRH matters.
Fasoranti, Onwuama [31]; 2018	Nigeria	To assess the access to health information and family influence on reproductive HL among school girls	Cross-sectional; quantitative	School; 243 female secondary school students aged 10–19 years; purposive sampling	Self-developed &pretested questionnaire	Access to reproductive health information is limited; the adolescents reproductive health literacy was poor, and family influenced the girls’ reproductive health literacy.
Obarisiagbon, Ofili [48]; 2019	Nigeria	To assess knowledge, attitude, preventive practices of HIV/AIDS and associated factors among school adolescents	Cross-sectional; quantitative	School; 440 secondary school students aged 10–19 years; stratified sampling	Pretested questionnaire	About 61.6% of the respondents had good knowledge of HIV/AIDS; 51.3% of the adolescents who had good knowledge had negative attitude towards HIV/AIDS, and the association between knowledge and attitude towards HIV/AIDS was significant (*p* = 0.01). About 73.5% of the sexually active adolescents had poor HIV/AIDS preventive practice.
Olaotan and Omokhodion [49]; 2020	Nigeria	To examine prevalence and predictors of unprotected sexual activities and abortion practices; access to and use of contraceptive services; and knowledge of and exposure to STIs among undergraduates	Cross-sectional; quantitative	College, University; 734 undergraduate students aged 17 to 24 years; multi-stage sampling/systematic sampling	Questionnaire	Many of the respondents reported being sexually active, and the abortion prevalence was high them. The different forms of contraception knowledge was poor. Males reported having better access to contraceptives than the females, and more males (36%) than females (6%) used contraceptives always during sexual intercourse.
Ramathuba, Khoza [50]; 2012	South Africa	To assess secondary school girls’ knowledge, attitudes and practices of contraception	Cross-sectional; quantitative	School; 273 secondary school girls; age range 13–19 years; convenience sampling	Questionnaire	The girls were aware of different contraceptive methods. However, ineffective and non- contraceptive use was reported due to pressure from male partners, fear of parental reaction to its use, reluctance to contraceptives use, poor education of contraceptives, and lack of counseling regarding the issue.
Mostert, Sethole [51]; 2020	South Africa	To assess technical school learners’ sexual knowledge and activities	Cross-sectional; quantitative	A rural technical secondary school; 79 learners in grade 8 to 12 aged 12 to 18; all learners from both genders were invited to participate	Questionnaire	About 24% of the participants engaged in early sexual activity, and the use of contraceptives was found to be low (41.2%). About 45.5% had never heard of STDs. Their primary source of information was reported as school-based programmes (58.0%).
Bankole, Biddlecom [52]; 2007	Sub-Saharan Africa (4 countries)	To assess adolescents’ sexual activity, knowledge about HIV, STIs and pregnancy prevention, and sources of information	Based on national surveys; quantitative	Based on national surveys of male and female 12–19 year olds; 8837 in number	Questionnaire	The adolescents reported already beginning to be sexually active and many of them believed that their close friends were sexually active. Although they reported having high levels of awareness, they had little in-depth knowledge about pregnancy and HIV prevention.
Ahinkorah, Hagan Jr [53]; 2020	Sub-Saharan Africa(32 countries)	To assess the relationship between female adolescents’ reproductive health decision-making capacity and their contraceptive utilisation	Based on DHS; Quantitative	Based on DHS that conducted in 32 countries in SSA; 15858 female adolescents (15–19 years old)	Based on DHS	About 68.7% found to have capacity to make reproductive health decisions, however, prevalence of contraceptive use was only 18.87%. Those who had the capacity to make reproductive health decisions had higher odds of using the contraceptives. The odds of using contraceptive increased with age of the adolescents. Also, contraceptive use increased with the level of education of the adolescents. Besides, the odds of contraceptive utilisation was reported as highest among those from the richest families, and those in rural areas reported lower contraceptives use.
Manda [54]; 2008	Tanzania	To assess students’ access to and use of SRH information services in universities in the context of gender dynamics and relations	Cross-sectional; mixed design	University; 194 undergraduate students; age range not clearly specified; convenience sampling & purposive sampling	Questionnaire, FGD guide, and key informant interview guide	Even though the students could access a wide range of sources of SRH information, the actual use was limited to radio, television, and friends, and information sources such as health workers and brochures/leaflets were reported as rarely used. Awareness of the SRH services availability was not wide spread among the students so that a large percentage of students think that they cannot access SRH information in the campuses, and they had negative attitude towards SRH information services provision in the campuses. However, SRH information access and use was not influenced by gender.
Mkumbo [55]; 2013	Tanzania	To assess school young people’s satisfaction level of current information sources and knowledge about sexual health	Cross-sectional; quantitative	Schools in rural and urban areas; 351 primary school students; aged 11–16 years; randomly selected	Questionnaire	More than 70% reported being most satisfied with sexual health information that they had received from health workers (and/or health facilities), other family members, and religious leaders, in that order. Less than 50% of reported being satisfied with the quality of sexual health information that they had got from their teachers. The majority reported not being satisfied with the quality of information they got from many of sexual health information sources they could access.
Mcharo, Mayaud [56]; 2021	Tanzania	To assess the experience and preferences of young people concerning SRH education and learning and communication with their parents or guardians	Cross-sectional; quantitative	Higher Learning Institutions (HLI); 504 students attending HLIs aged 18–24 years; randomly selected	Questionnaire	About 88.5% of the respondents were sexually active. 61% reported not discussing SRH matters with their parent or guardian. 30.2% and 22.7% reported learning about SRH matters from peers and teacher/school respectively. Females and males reported preferring discussions with adults of their respective sexes. Peers, media, and schools were reported as the preferred SRH information sources (18.2%, 16.2%, and 14.2% respectively).
Jones and Norton [57]; 2007	Uganda	To assess the school girls’ sexual health information sources, freedom of choice and sexual decision-making, and opportunities to embrace their sexual right and make informed sexual choices	Ethnographic study; qualitative	School in rural area; 13 secondary school girls; approximately 17 years of age; all female students in grade 10	Interview guide, observations guide, and document analysis	Although the girls were well-informed about the risks and responsibilities of sexual activity, problems such as poverty and sexual abuse constrained their options. Even if they might be believed in abstaining from sex until marriage, they engaged in sex to get income and pay for school, clothing, and others. Fear of sexual abuse, early/unintended pregnancy, and STDs constrained attempts to embrace their sexuality.
Kemigisha, Bruce [58]; 2018	Uganda	To assess the very young adolescents’ SRH knowledge, information-seeking, and sexual behavior	Cross-sectional; quantitative	Schools in rural and urban areas; 1096 young adolescents in primary schools aged 10–14 years; systematic sampling	Standardised questionnaire	About 95% knew HIV, while only 37% knew other STIs, and 56% mentioned at least one modern method that help to prevent pregnancy. About 85% reported that they access SRH information in the media; 31% mentioned a school as source; while only 10% and 22% reported consulting their father and mother respectively. About 7.6% had ever had sexual intercourse, and 90% of them were not using any protection.
Ivanova, Rai [59]; 2019	Uganda	To assess refugee girls’ SRH experiences, knowledge and access to services in a settlement	Cross-sectional (mixed approach)	Nakivale refugee settlement; 260 refugee girls aged 13–19 years; convenience sampling; purposive sampling	Questionnaire and interview guide	About 11.7% reported not being aware of how HIV is prevented; 15.7% reported not knowing any STI, and 13.8% reported not familiar with any pregnancy prevention method. They reported parents or guardians as the most preferred sources for SRH information, though they reported being afraid of discussing sexuality topics except menstruation with their parents.
Namukonda, Rosen [60]; 2021	Zambia	To assess school adolescents’ SRH knowledge, attitudes and service utilisation experiences in the context of CSE implementation	Cross-sectional; mixed approach	School; 1,612 in-school young people aged 12–24 years; randomly surveyed	Questionnaire; FGD guide	Only less than half of sexually experienced adolescents reported accessing SRH services in spite of having moderate SRH knowledge and acceptability to SRH services. Factors influencing SRH service uptake, include low perceived benefits of the services, unsupportive parental and community environments, and unfriendly environment or interactions with healthcare providers.
Moyo and Rusinga [61]; 2017	Zimbabwe	To assess knowledge, attitudes, beliefs and practices regarding contraceptive use among adolescents and to understand reproductive health education importance for the adolescents in using contraceptives	Cross-sectional; mixed methods	Community-based; 185 adolescents aged 15–19 years; systematic sampling	Questionnaire; FGD, key informant interview guides	Early sexual debut engagement was common among the adolescents. Although knowledge regarding modern contraceptives is common (96%) among them, contraceptive use was very low, 21%, due to different factors, including demographic, policy, socio-cultural, religious, and economic ones.

## Results

The searches yielded a total of 2,682 studies. After removing 61 duplicates, the titles and abstracts of the remaining 2,621 studies were screened for relevance. Of these, 99 studies were selected for further assessment. Out of the 99 articles, 24 met the inclusion criteria, while 75 did not and were excluded with reasons (see Supplementary Material). The reference lists of the 24 selected studies were screened for additional relevant studies; however, no additional relevant study was found. Thus, only 24 studies met the eligibility criteria and were included in this study. Overviews of these eligible studies are provided in Table 1.

## Characteristics of the eligible studies

The majority of eligible studies were conducted in six sub-Saharan African countries, namely, Ethiopia, Ghana, Nigeria, South Africa, Tanzania, and Uganda. Most of these studies were published within the last decade and the last 2 years, with the oldest dating back to 2003 [40]. All of the included studies were cross-sectional, with the exception of four studies, one of which [45] was a quasi-experimental study, another [57] was a longitudinal ethnographic study, and the remaining two studies [52] and [53] were based on nationally representative household surveys and DHS. Seven of the studies were mixed-methods studies [40,41,43,54,59–61], one study was a qualitative study [57], and the remaining 16 studies were quantitative studies.

Two of the eligible studies [41,61] were community-based, two [52,53] were secondary data-based, and two [42,59] were based on young people with disability associations and refugee settlement, respectively. The remaining 18 studies were school/college/university-based. Five of the included studies [31,46,50,54,59] employed different types of non-probability sampling techniques. In three studies [39,51,57], all members of the studies populations were participants. Fourteen of the eligible studies employed different types of probability sampling techniques, while the sampling techniques employed in the remaining two studies [44,45] were not clearly specified.

In the majority of the included studies, participants were in their teens; in some studies, the age of participants ranged up to 24 years, and in two studies [47,54], the age ranges of participants were not clearly specified, but they were undergraduate students and in-school adolescents, respectively. As tools of quantitative data collections, three studies [39,49,56] used different instruments that were adapted from different validated questionnaires. One study [51] used a questionnaire that was developed based on literature and input from stakeholders. Similarly, Fasoranti et al. [31] used a self-developed and validated questionnaire. Five studies [40–43,46] mentioned that they used pre-tested questionnaires. The remaining studies [44,45,47,48,50,52–55,58–61] did not specify whether the questionnaires were validated or pretested.

## Findings of the eligible studies

Findings of the eligible studies are presented by categorising similar issues including the SRH-related competencies, practices, and experiences of the studies participants.

## SRH knowledge among young people

Of the 24 eligible studies, 20 studies measured SRH knowledge of participants. Of these 20 studies, 13 studies were concerned with young people in schools, and seven of these studies [44,50–52,55,58,60] found that the majority of respondents had inadequate SRH knowledge, while the remaining six studies [39,40,45,47,48,57] reported that more than half of respondents had good knowledge of some aspects of SRH. For instance, Barchi et al. [39] found that 90% of respondents were aware of one or more forms of contraception, although only 67% could name a method for which they knew a source, and Obarisiagbon et al. [48] reported that 61.6% of study participants had good knowledge of HIV/AIDS. Three of the 20 studies measured SRH knowledge among young people at universities, and one of them [49] found poor SRH knowledge among participants, while the remaining two studies [43,46] found good knowledge levels in different aspects of SRH among respondents. For instance, Yared et al. [43] reported that most (89.4%) of the participants knew of modern contraceptives such as pills (64.8%) and condoms (56.8%).

Three studies [41,42,59] that included a study on young people with disabilities, a study on refugee girls in a settlement, and a rural community-based study on adolescents, respectively, found low SRH knowledge among the respondents. The remaining study, a community-based study [61], reported finding good SRH-related knowledge among the study participants. The study found that knowledge about modern contraceptives was universal (96%) among the respondents, although only 21% of the respondents translated this knowledge into practice. In general, more than half of the studies that measured SRH knowledge among young people found that the respondents had inadequate knowledge; however, others found that the participants had good knowledge in some aspects of SRH, such as HIV/AIDS, modes of transmission and prevention of STD/STIs, modern contraceptives, and where to obtain contraceptive services.

## SRH information sources and use among young people

Of the total eligible studies, 16 studies identified different sources of SRH information and knowledge among young people. Of the 16 studies, 13 studies [39,40,47,48,50–52,55,57,58] were conducted on young people in school settings, and three studies [43,54,56] were conducted on young people in university settings. These studies identified radio, television, friends or peers, teachers/school-based lessons or programmes, family members other than parents (e.g. older sisters, parental aunts for girls), social media/internet, health workers, parents, newspapers, magazines, books, and religious leaders or religious teachings as sources of SRH information among young people. However, as these studies reported, there was a variation in the extent of access to and use of these sources of information, with some being more accessible and utilised than other sources among young people. Peers or friends, media, and teachers/school lessons or programmes were the most widely used SRH information sources among young people.

As reported in many of these studies [39,50–52,56–58], parents were not widely used as a source of SRH information among young people. For instance, in a study on a very young people in school, Kemigisha et al. [58] reported that media (radio or television) (85%) was the most-utilised source of SRH information, while parents (mother (22.3%) and father (9.9%)) were the underutilised sources of SRH information among the study participants. Health professionals or health facilities were also reported as underutilised sources of SRH information among young people in many of the studies. However, in a study conducted on young people in urban and rural areas, Mkumbo [55] reported that the majority (about 70%) of the study participants were more satisfied with the quality of SRH information and knowledge from the sources they reported receiving relatively little information, such as health workers or health facilities and religious teachings, than the information they received from other sources, such as peers or friends. Likewise, in a study on school adolescents, Barchi et al. [39] found that adolescents who got SRH information from health professionals benefited more than those who got SRH information from other sources.

The remaining three studies [42,59,61] (i.e. a community-based study, a study conducted on young people with disabilities, and a study conducted on refugee girls in a settlement, respectively) also identified multiple sources of SRH information among young people, including those mentioned earlier. However, as in the above studies, health facilities/professionals and parents were under-utilised as source of SRH information. For example, in a community-based study, Moyo and Rusinga [61] reported that many adolescents sought advice about SRH from peers, while parents, teachers, health workers, and religious leaders had very little input on the issue. Similarly, in a study on young people with disabilities, Kassa et al. [42] found that the majority (about 78%) did not have discussions on SRH issues with their parents. The main sources of SRH information among the respondents were in the order of television/radio, friends, school teachers, and health professionals. Additionally, a number of the eligible studies, e.g [31,40,42,47,54], reported that young people have low access to SRH information and services.

## SRH-related experiences among young people

Of the total eligible studies, 17 studies [39–46,48–52,57–61] reported on SRH-related experiences, including sexual activities, protection use, and outcomes among young people. A number of or many of the participants in these studies reported engaging in sexual activity, even at early ages. Most sexually active participants in studies of adolescents in schools [40,48,50,51,57,58,60] and young people at universities [43,46,49] exhibited poor STDs prevention practices, either due to a lack of knowledge or despite having awareness and knowledge of the issue.

For instance, in a study on very young adolescents in school, Kemigisha et al. [58] reported that the adolescents lacked detailed SRH knowledge on types of STDs, HIV transmission, and contraception, but 7.6% of them were sexually active, of whom 90% were not using any protection. In a study on university students, Yared et al. [43] found that almost all of the respondents were aware of STIs (94.5%) and HIV/AIDS (98%), and most of the participants knew about protection methods. However, it was reported that more than half of the study participants had ever had sexual intercourse, and there were high rates of unwanted pregnancy and unsafe abortion, and more than 22.8% of them had had STIs in the past one year.

Similarly, a community-based study [61], a study conducted on young people with disabilities [42], and a study conducted on refugee girls in a settlement [59] found that information and knowledge about SRH did not translate into preventive practices among the participants, in addition to the knowledge gap problem. For instance, Moyo and Rusinga [61] found that nearly all study participants (96%) had knowledge of modern contraceptives, and 37% of the participants were sexually active, but 79% of the sexually active participants had never used any form of contraception.

Thus, according to these studies, many sexually active young people were vulnerable to or victims of STIs, STDs, and unplanned pregnancy. As Yared et al. [43] and Molla et al. [41] reported, many of those who were exposed to STIs/STDs or unplanned pregnancy did not seek care or services from any source or sought traditional medicine or practised life-threatening abortions.

## Factors influencing SRH-related practices and experiences among young people

Of the total eligible studies, 17 studies assessed and identified factors determining SRH-related practices and experiences among young people, including SRH-related information and service access and use, sexual activities, and outcomes. As reported in studies conducted on young people in schools [31,39,40,45,47,48,50,57,60], the influencing factors include family influence, economic status, power differentials between males and females, sex, age, grade/education level, access to health facilities, peer influence, lack of commitment, access to health education/information, and cultural values and norms. For example, Ramathuba et al. [50] reported that in a study on young school girls, although 75% of the girls had knowledge about the different contraceptive methods, many of the girls (about 42%) who were sexually active did not protect themselves from STIs/STDs. This study identified pressure from male partners, fear of parental reaction, reluctance, poor education, and lack of counselling as the main causes for ineffective use or non-utilisation of protections among the girls.

Studies conducted on young people at universities [46,49,54], in addition to the aforementioned related ones, identified time (workload), user-friendly services, and faculty of study as factors influencing SRH-related practices and experiences among young people. Community-based studies [41,61], a study on young people with disabilities [42], a study on refuge girls in a settlement [59], and a study based on DHS [53] also identified different factors influencing SRH-related practices and experiences among young people. These factors include the aforementioned factors, as well as decision-making capacity and residence (rural/urban) [53], and impairment/disability type [42].

## Discussions and implications

The aim of this review was to provide a coherent summary and synthesis of existing literature on SRHL among young people in SSA, and to indicate the implications for policy, interventions, and future research. As the review findings indicate, rather than directly addressing (the concept of) SRHL, the eligible studies were mostly concerned with only some aspects/elements of SRHL, focusing on SRH knowledge, practices, and experiences among young people. The included studies examined SRH knowledge [39–52,55,57–61], sources of SRH information and knowledge [39,40,42,43,47,48,50–52,54–59,61], SRH-related experiences, including sexual activities, protection use, and outcome [39–46,48–52,57–61], and factors determining SRH-related practices and experiences, such as SRH-related information and service access and use, sexual activities, and outcomes [31,39–42,45–48,50,53,54,57,59,60] among young people in school, university, and other different contexts.

Although most of the studies on SRH knowledge among young people were conducted on students in school and university settings, more than half of these studies reported inadequate SRH knowledge among the respondents. As evidence indicates, even though knowledge is not sufficient in itself, it is often a necessary factor for healthy sexual and reproductive behaviours or practices [62,63]. Poor or inadequate SRH knowledge may lead to poor SRH practices among young people [63,64]. Thus, according to many of the eligible studies, the majority of the studies participants were vulnerable to SRH-related problems due to a lack of adequate SRH knowledge and understandings.

The eligible studies identified multiple sources of SRH information and knowledge among young people in different settings. In many of these studies, peers/friends, media, and schools were identified as more widely used sources of SRH information than other sources among young people. Most studies reported that parents did not serve as a primary SRH information source for the majority of participants. Additionally, in many of these studies, young people reported low utilisation of health facilities or health professionals as sources of SRH information. Similarly, as stated by a number of studies, for instance, Masemola-Yende and Mataboge [65], young people, specifically, females, prefer obtaining SRH information from their peers/friends because they feel more comfortable discussing SRH matters with them than with their parents or healthcare providers. However, as reported in a number of eligible studies, young people were more satisfied with the quality of SRH information they received from health professionals than from the other sources.

Studies, for instance [66–68], have noted that in the presence of many alternative sources of SRH information for young people, it may be difficult for them to determine which sources are accurate and which are not. Much of the SRH information that young people are exposed to, especially, media may be inaccurate or misleading [47]. Parents could play a critical role in mitigating the potential harm of inappropriate information sources [68,69] and in advising their adolescent children about issues related to SRH [68,70,71]. However, as many of the eligible studies reported, young people’s discussions with their parents about the issue are limited or absent. Likewise, although school-based SRH-related education programmes could play a great role in this regard [64,72], it appears from the eligible studies that not many young people have accessed such opportunities. Thus, as noted in a number of the included and other related studies, such as [61,65], in the absence of open discussions with parents and the absence of or low access to accurate SRH information, young people depend on advices/information from their ill-informed peers or friends and their own personal experiments. They also become vulnerable to misleading information from the media [47,67] and are exposed to various SRH-related problems, as reported in a number of the reviewed/eligible studies.

The included studies reported that young people, including very young adolescents in school, had already become sexually active. However, as stated in those studies, there were poor preventive practices or poor utilisation of health services among the majority of the sexually active young people. Consequently, many were exposed to STIs and/or unplanned pregnancy, and many of those who were exposed to these problems did not seek healthcare services. As evidence indicates, young people often make SRH decisions mainly based upon their knowledge and sources of information, in addition to the behaviours of their peers [73,74]. However, a number of eligible studies reported that there were problems with putting SRH knowledge into actual practices among young people who were sexually active, in addition to a lack of knowledge and understanding among many about the issue. Also, as noted in many other studies, for instance [75,76], the problem of not exercising the knowledge among young people is widespread. Like in the case of lack of knowledge, the disconnect between SRH knowledge and actual practice is a critical problem that puts young people at risk of SRH problems, as was also reported in a number of eligible studies.

The included studies identified diverse factors that determine SRH-related practices and experiences among young people. These factors include family influences, peer pressures, access to SRH information and services, economic circumstances, culture or norms, social position, time (workload), user-friendly services, residence place, age, sex, education, commitment, and health status. This indicates that SRHL among young people is affected by a wide range of factors, from personal to broader structural conditions. The literature also indicates that HL, including SRHL, is determined by multiple factors [4,21,27]. Therefore, although providing SRH information and health education interventions for young people is a fundamental measure for improving their SRH [62–64,72], such measures may not be successful on their own. As noted in many of the eligible studies and other related literature, such interventions may only help young people acquire SRH information and knowledge, without necessarily enabling them, especially girls, to make appropriate SRH decisions, choices, and practices due to the above-listed related influencing/hindering factors.

To summarise, the review findings indicate that the eligible studies participants had low SRHL-related skills. The review identified a wide range of SRH information sources for young people, but access to and use of these sources varied. The review revealed that many young people with poor SRH knowledge were already engaging in sexual activities that placed them at risk for STIs and teenage pregnancy. Additionally, the review found that many young people with good SRH knowledge did not put their knowledge into action. Finally, the review identified diverse personal, sociocultural, and economic factors that influence SRHL among young people. Based on these findings, the implications for policy, interventions, and future research are provided as follows:

- Inadequate SRHL among young people needs to be addressed. Addressing this problem requires ensuring the establishment and implementation of policies concerning the issue. It requires expanding SRH information and services access and tailoring SRH information content for each specific group of young people. It also requires fostering cooperation among all stakeholders, including policy-makers, health professionals, schools, media, religious organisations, parents, and others.
- Early sexual activities, teenage pregnancy, and STIs problems among young people call for placing a strong emphasis on the development or reinforcement of SRH education programmes for schools and communities. Schools need to incorporate SRH education and skills building into their curricula to promote SRHL among young people. Teachers need training to provide accurate and adolescent-friendly SRH information services, and to promote active discussions on the issue among students. Communities need SRH education programmes to enable parents, religious bodies, and community figures to communicate effectively with and educate young people about SRH issues. Parents and religious figures need to communicate actively with young people and provide them with appropriate SRH information.
- The problem of a disconnect between SRH knowledge and practice, as well as poor SRH-related knowledge and practice among sexually active young people, call for the introduction and/or implementation of sustained behaviour change programmes with group-specific and accurate SRH education to promote the desired behavioural changes among them. These programmes need to be inclusive, sustainable, and young people-friendly.
- Comprehensive SRH education may help delay the onset of sexual practices among young people if delivered before they become sexually active [58,77]. Therefore, young people need to receive comprehensive SRH education tailored to their age before they engage in sexual activity. This education should provide young people with detailed SRH information, including the risks of early sexual activities or having sex before marriage and the benefits of waiting. The provision of such information needs to be routine in families, religious institutions, schools, and youth associations/clubs to enable young people make informed SRH decisions and practice healthy lifestyles throughout their lives.
- The SRH programmes must fully understand, acknowledge, and address the multiple factors that influence young people’s SRHL and SRH decisions and practices. They must be designed, implemented, and evaluated in cooperation with all stakeholders, including the full engagement of young people themselves to meet the specific needs of and foster SRHL in young people.
- A deeper understanding of SRHL in young people is essential to develop effective interventions to improve SRHL and SRH outcomes among them. Therefore, SRHL studies among each audience of young people within different circumstances are needed.

## Strengths and limitations

This review has provided a coherent summary and synthesis of available literature on SRHL among young people in SSA, with important implications for policy, interventions, and future research. However, this review may have some limitations due to the following points. Quality assessment of the included studies was not conducted with the intention of getting more articles, based on the purpose of the review. Evidence for this review was retrieved from PubMed, CINAHL, AJOL, AIM, and Google Scholar; thus, relevant studies from other databases may have been missed. The eligible studies included different groups of participants with different age ranges (between 10 and 24 years); thus, comparison of SRHL experiences across the studies may be difficult. The studies included in this review were mostly concerned with only some elements of SRHL, as there were no studies that directly measured the issue or all its aspects among the target group. Only papers written in English were eligible for this review; therefore, other relevant studies written in other languages may have been missed.

## Conclusion

The review found that studies on SRH literacy among young people in sub-Saharan Africa are limited. The available evidence indicates that SRH literacy among young people in sub-Saharan Africa is concerning and has not been fully researched. SRH literacy studies are needed to better understand the issue, and evidence-based interventions are needed to improve SRH literacy and health outcomes among young people.

## Supplementary Material

Supplemental Material

## Paper context

Sexual and reproductive health literacy is a key to achieving sexual and reproductive health, especially among young people in low-income countries, such as sub-Saharan Africa. As this review indicates, sexual and reproductive health literacy among young people in sub-Saharan Africa is problematic and has not been fully researched. A deeper understanding of the issue is essential to design and implement effective interventions to improve sexual and reproductive health literacy and health outcomes among young people.
