# Supplementary material for: Sexual and reproductive health literacy among young people in Sub-Saharan Africa: evidence synthesis and implications
Source: Glob Health Action. 2023 Nov 27;16(1):2279841. doi: 10.1080/16549716.2023.2279841 (PMC10795590; doi:10.1080/16549716.2023.2279841)
Supplement: Supplemental Material [file ZGHA_A_2279841_SM9192.docx]

**Supplementary Materials**

**Supplementary S1: Search strategies and searching date for the databases searched**

| Search strategy | Date searched | Search No. | Query/search | Result |
| --- | --- | --- | --- | --- |
| PubMed | 18/03/2022 | 1 | (((("Reproductive health literacy" [tw]) OR ("Sexual health literacy"[tw])) OR (Sexual AND reproductive "Health literacy" [Mesh])) OR (Sexual AND reproductive "Health literacy"[tw])) OR (("Reproductive Health"[Mesh] OR "Reproductive Health"[tiab]) OR ("Sexual Health"[Mesh] OR "Sexual Health"[tiab])) (Filters applied :Full text, English) | 24,268 |
|  |  | 2 | (((((("Adolescent"[Mesh]) OR (Adolscen*)) OR ("Young Adult"[Mesh])) OR (Young*)) OR (Teen*)) OR (Student*)) OR (Youth) (Filters applied :Full text, English) | 2,690,471 |
|  |  | 3 | **(("Africa South of the Sahara"[Mesh]) OR (“Sub-Saharan Africa” [tiab])) OR (Angola[tiab] OR Benin[tiab] OR Botswana[tiab] OR "Burkina Faso"[tiab] OR Burundi[tiab] OR "Cabo Verde"[tiab] OR Cameroon[tiab] OR "Central African Republic"[tiab] OR Chad[tiab] OR Comoros[tiab] OR "Democratic Republic of the Congo"[tiab] OR "Republic of the Congo"[tiab] OR "Cote D'ivoire"[tiab] OR "Equatorial Guinea"[tiab] OR Eritrea[tiab] OR Eswatini[tiab] OR Ethiopia[tiab] OR Gabon[tiab] OR "Gambia, THE"[tiab] OR Ghana[tiab] OR Guinea[tiab] OR "Guinea-Bissau"[tiab] OR Kenya[tiab] OR Lesotho[tiab] OR Liberia[tiab] OR Madagascar[tiab] OR Malawi[tiab] OR Mali[tiab] OR Mauritania[tiab] OR Mauritius[tiab] OR Mozambique[tiab] OR Namibia[tiab] Or Niger[tiab] OR Nigeria[tiab] OR Rwanda[tiab] OR "Sao Tome and Principe"[tiab] OR Senegal[tiab] OR Seychelles[tiab] OR "Sierra Leone"[tiab] OR Somalia[tiab] OR "South Africa"[tiab] OR "South Sudan"[tiab] OR Sudan[tiab] OR Tanzania[tiab] OR Togo[tiab] OR Uganda[tiab] OR Zambia[tiab] OR Zimbabwe[tiab])** (Filters applied :Full text, English) | 200,428 |
|  |  | 4 | ((#1) AND (#2)) AND (#3) (Filters applied :Full text, English) | 1,920 |
| CINAHL | 25/03/2022 | S1 | (MH "Health Literacy") OR (MH "Reproductive Health") OR "Reproductive health literacy" OR (MH "Health Literacy") OR (MH "Sexual Health") OR "Sexual health literacy" | 19,596 |
|  |  | S2 | (MH "Adolescence+") OR "Adolescence" OR (MH "Young Adult") OR "Young adult" OR (MH "Students+") OR "Students" OR (MH "Students, High School") OR (MH "Students, College") OR (MH "Students, Middle School") | 865,945 |
|  |  | S3 | (MH "Africa South of the Sahara+") OR (MH "Africa, Western+") OR (MH "Africa, Southern+") OR (MH "Africa, Eastern+") OR (MH "Africa, Central+") OR "Sub-Saharan Africa" | 78,486 |
|  |  | S4 | S1 AND S2 AND S3 | 735 |
| AJOL | 28/03/2022 |  | Sexual and reproductive health literacy | 6 |
| AIM | 04/04/2022 |  | Sexual and reproductive health literacy | 4 |
| Google Scholar | Up to May 20, 2022 |  | Sexual and reproductive health literacy and sub-Saharan Africa | 17 |

**Supplementary S2: Data extraction tool**

| **Paper/study information** | |
| --- | --- |
| Title of the study |  |
| Author(s) of the study |  |
| Year of its publication |  |
| Country where the study was conducted |  |
| Study purpose |  |
| Study setting |  |
| Type of the study (study design/method) |  |
| Study population |  |
| Participants selection process |  |
| Details about participants ( age, gender, and number) |  |
| Instrument utilized |  |
| Major findings of the study |  |

**Supplementary S3: Studies excluded with reasons (n=75)**

**Focused on only SRH Knowledge (n=22)**

1. ADINEW, Y. M., WORKU, A. G. & MENGESHA, Z. B. 2013. Knowledge of reproductive and sexual rights among University students in Ethiopia: institution-based cross-sectional. BMC international health and human rights, 13**,** 1-7.
2. AKUIYIBO, S., ANYANTI, J., IDOGHO, O., PIOT, S., AMOO, B., NWANKWO, N. & ANOSIKE, N. 2021. Impact of peer education on sexual health knowledge among adolescents and young persons in two North Western states of Nigeria. Reproductive Health, 18**,** 1-8.
3. AMUYUNZU-NYAMONGO, M., BIDDLECOM, A. E., OUEDRAOGO, C. & WOOG, V. 2005. Qualitative evidence on adolescents’ views of sexual and reproductive health in Sub-Saharan Africa. Occasional Report, 16.
4. AREDO, M. A., SENDO, E. G. & DERESSA, J. T. 2021. Knowledge of cervical cancer screening and associated factors among women attending maternal health services at Aira Hospital, West Wollega, Ethiopia. SAGE open medicine, 9**,** 20503121211047063.
5. AYALEW, M., NIGATU, D., SITOTAW, G. & DEBIE, A. 2019. Knowledge and attitude towards sexual and reproductive health rights and associated factors among Adet Tana Haik College students, Northwest Ethiopia: a cross-sectional study. BMC Research Notes, 12**,** 1-7.
6. ADEBOMI, O. & OLUFEMI, A. 2013. Sources of Reproductive Health Information and Attitude towards Reproductive Health Practices among Female Adolescents with Hearing Impairment in Lagos and Oyo states, Nigeria. AFRICAN JOURNAL FOR THE PSYCHOLOGICAL STUDIES OF SOCIAL ISSUES, 16**,** 28-36.
7. BEKELE, D., SURUR, F., NIGATU, B., TEKLU, A., GETINET, T., KASSA, M., GEBREMEDHIN, M., GEBREMICHAEL, B. & ABESHA, Y. 2020. Knowledge and attitude towards family planning among women of reproductive age in emerging regions of Ethiopia. Journal of Multidisciplinary Healthcare, 13**,** 1463.
8. BELETEW ABATE, B., HABTAMU GELAW, K., FENTAW, H., ASHAGIRE, M. & MEKASH, T. 2020. Knowledge Level and Associated Factors of Reproductive Health Issues among Secondary School Students in Woldia Town, Amhara, Ethiopia, 2019: A Cross-Sectional Study. Journal of Environmental and Public Health, 2020.
9. CHALI, K., OLJIRA, D., SILESHI, T. & MEKONNEN, T. 2021. Knowledge on cervical cancer, attitude toward its screening, and associated factors among reproductive age women in Metu Town, Ilu Aba Bor, South West Ethiopia, 2018: community‐based cross‐sectional study. Cancer Reports, 4**,** e1382.
10. EGEMBA, M. & AJUWON, A. 2015. Knowledge and perceptions of reproductive rights among female postgraduate students of the University of Ibadan, Nigeria. African Journal of Biomedical Research, 18**,** 95-107.
11. FINLAY, J. E., ASSEFA, N., MWANYIKA‐SANDO, M., DESSIE, Y., HARLING, G., NJAU, T., CHUKWU, A., ODUOLA, A., SHAH, I. & ADANU, R. 2020. Sexual and reproductive health knowledge among adolescents in eight sites across sub‐Saharan Africa. Tropical Medicine & International Health, 25**,** 44-53.
12. GEREMEW, A. B., GELAGAY, A. A. & AZALE, T. 2018. Comprehensive knowledge on cervical cancer, attitude towards its screening and associated factors among women aged 30–49 years in Finote Selam town, northwest Ethiopia. Reproductive health, 15**,** 1-12.
13. GETAHUN, F., MAZENGIA, F., ABUHAY, M. & BIRHANU, Z. 2013. Comprehensive knowledge about cervical cancer is low among women in Northwest Ethiopia. BMC cancer, 13**,** 1-7.
14. GOVENDER, D., NAIDOO, S. & TAYLOR, M. 2019. Knowledge, attitudes and peer influences related to pregnancy, sexual and reproductive health among adolescents using maternal health services in Ugu, KwaZulu-Natal, South Africa. BMC Public Health, 19**,** 1-16.
15. MAKINDE, O. A. & ADEBAYO, A. M. 2020. Knowledge and perception of sexual and reproductive rights among married women in Nigeria. Sexual and Reproductive Health Matters, 28**,** 1731297.
16. MBUGUA, S. M. & KARONJO, J. M. 2018. Reproductive health knowledge among college students in Kenya. BMC public health,18**,** 1-7.
17. NDONGMO, T. N., NDONGMO, C. B. & MICHELO, C. 2017. Sexual and reproductive health knowledge and behavior among adolescents living with HIV in Zambia: a case study. The Pan African Medical Journal, 26.
18. NWAORGU, O. C., ONYENEHO, N. G., OKOLO, M., OBADIKE, E. & ENIBE, G. 2008. Reproductive health knowledge and practices among junior secondary school grade one students in Enugu State: threat to achieving millennium development goals in Nigeria.
19. OYO-ITA, A., KALU, Q., MKPANAM, N., IKPEME, B. & ETUK, S. 2004. Knowledge of reproductive health issues among secondary school adolescents in Calabar, Nigeria. Global Journal of Medical Sciences, 3**,** 5-8.
20. TEGEGN, A., YAZACHEW, M. & GELAW, Y. 2008. Reproductive health knowledge and attitude among adolescents: a community based study in Jimma Town, Southwest Ethiopia. The Ethiopian Journal of Health Development, 22.
21. WAKWOYA, E. B., GEMECHU, K. S. & DASA, T. T. 2020. Knowledge of cervical cancer and associated factors among women attending public health facilities in Eastern Ethiopia. Cancer Management and Research, 12**,** 10103.
22. WOLDU, B. F., LEMU, L. G. & MANDARO, D. E. 2020. Comprehensive knowledge towards cervical cancer and associated factors among women in Durame town, Southern Ethiopia. Journal of cancer epidemiology, 2020.

**Focused on only SRH services utilization (n=11)**

1. ABATE, A. T. & AYISA, A. A. 2019. Reproductive health services utilization and its associated factors among secondary school youths in Woreta town, South Gondar, North West Ethiopia: a cross sectional study. BMC research notes, 12**,** 1-7.
2. BINU, W., MARAMA, T., GERBABA, M. & SINAGA, M. 2018. Sexual and reproductive health services utilization and associated factors among secondary school students in Nekemte town, Ethiopia. Reproductive health, 15**,** 1-10.
3. BIRHAN, Z., TUSHUNE, K. & JEBENA, M. G. 2018. Sexual and reproductive health services use, perceptions, and barriers among young people in southwest Oromia, Ethiopia. Ethiopian journal of health sciences, 28**,** 37-48.
4. BWAMBALE, M. F., BUKULUKI, P., MOYER, C. A. & VAN DEN BORNE, B. H. 2021. Utilisation of sexual and reproductive health services among street children and young adults in Kampala, Uganda: does migration matter? BMC health services research, 21**,** 1-9.
5. GEBREYESUS, H., TEWELDEMEDHIN, M. & MAMO, A. 2019. Determinants of reproductive health services utilization among rural female adolescents in Asgede-Tsimbla district Northern Ethiopia: a community based cross-sectional study. Reproductive health, 16**,** 1-10.
6. GURARA, A. M., GIZAW, A. B., AYALEW, A. K. & KEDIRO ADEM, A. 2020. Reproductive health service utilization and associated factors among adolescents at public school in Adama town east Shewa Ethiopia, 2018. Nursing Practice Today, 7**,** 275-285.
7. KASIM, J., KALU, A., KAMARA, B. & ALEMA, H. B. 2020. Cervical cancer screening service utilization and associated factors among women in the Shabadino District, Southern Ethiopia. Journal of Cancer Epidemiology, 2020.
8. LIYEH, T. M., GOSHU, Y. A., BELAY, H. G., TASEW, H. A., MIHIRETIE, G. N. & AYALEW, A. B. 2021. Youth Reproductive Health Service Utilization and Associated Factors among Amhara Region Female Night Students, Ethiopia. BioMed Research International, 2021.
9. ODO, A. N., OFUEBE, J. I., ANIKE, A. I. & SAMUEL, E. S. 2021. Predictors of young people’s use of sexual and reproductive health services in Nigeria: a mixed-method approach. BMC public health, 21**,** 1-8.
10. SHABANI, O. & TSHITANGANO, T. G. 2019. Determinants of the utilisation of sexual and reproductive healthcare services by male adolescents in the Tshwane Metropolitan Municipality in South Africa. Health SA Gesondheid, 24**,** 1-8.
11. TLAYE, K. G., BELETE, M. A., DEMELEW, T. M., GETU, M. A. & ASTAWESEGN, F. H. 2018. Reproductive health services utilization and its associated factors among adolescents in Debre Berhan town, Central Ethiopia: a community-based cross-sectional study. Reproductive health, 15**,** 1-11.

**Focused on only accessing SRH services (n=6)**

1. ARULOGUN, O. S., TITILOYE, M. A., AFOLABI, N. B., OYEWOLE, O. E. & NWAORGU, O. G. 2013. Experiences of girls with hearing impairment in accessing reproductive health care services in Ibadan, Nigeria. African Journal of Reproductive Health, 17**,** 85-93.
2. HAILEMARIAM, S., GUTEMA, L., AGEGNEHU, W. & DERESE, M. 2021. Challenges faced by female out-of-school adolescents in accessing and utilizing sexual and reproductive health service: a qualitative exploratory study in Southwest, Ethiopia. Journal of Primary Care & Community Health, 12**,** 21501327211018936.
3. IBEGBULAM, I. J., AKPOM, C. C., ENEM, F. N. & ONYAM, D. I. 2018. Use of the Internet as a source for reproductive health information seeking among adolescent girls in secondary schools in Enugu, Nigeria. Health Information & Libraries Journal, 35**,** 298-308.
4. KACHOTA, B. J. & KASSIM, M. 2021. Sexual and reproductive health information-seeking behaviour of undergraduate students at Mzumbe University, Morogoro–Tanzania. University of Dar es Salaam Library Journal, 16**,** 115-130.
5. TEGEGN, A. & GELAW, Y. 2009. Adolescent reproductive health services in jimma city: accessibility and utilization. Ethiopian Journal of Health Sciences, 19.
6. TILAHUN, T., BEKUMA, T. T., GETACHEW, M. & SEME, A. 2021. Assessment of access and utilization of adolescent and youth sexual and reproductive health services in western Ethiopia. Reproductive Health, 18**,** 1-9.

**Focused on only SRH communication (n=5)**

1. AYEHU, A., KASSAW, T. & HAILU, G. 2016. Young people’s parental discussion about sexual and reproductive health issues and its associated factors in Awabel woreda, Northwest Ethiopia. Reproductive health, 13**,** 1-8.
2. DAGNACHEW ADAM, N., DEMISSIE, G. D. & GELAGAY, A. A. 2020. Parent-Adolescent Communication on Sexual and Reproductive Health Issues and Associated Factors among Preparatory and Secondary School Students of Dabat Town, Northwest Ethiopia. Journal of Environmental and Public Health, 2020.
3. GEZAHEGN, T., BIRHANU, Z., AMAN, M., DESSALEGN, M., ABERA, A. & NYAGERO, J. 2016. Peer communication on sex and sexual health among youths: a case of Debre Berhan university, Ethiopia. The Pan African Medical Journal, 25.
4. MELAKU, Y. A., BERHANE, Y., KINSMAN, J. & REDA, H. L. 2014. Sexual and reproductive health communication and awareness of contraceptive methods among secondary school female students, northern Ethiopia: a cross-sectional study. BMC public health, 14**,** 1-11.
5. TSAKANI, L. R., DAVHANA-MASELESELE, M. & OBI, L. C. 2011. Teenagers’ experiences of sexual health dialogue in the rural villages of the Vhembe District, Limpopo Province. Health SA Gesondheid, 16.

**Focused on SRH services needs (n=3)**

1. ABABOR, A. A., TESSO, D. W. & CHEME, M. C. 2019. Addressing the deprived: need and access of sexual reproductive health services to street adolescents in Ethiopia. The case of Nekemte town: mixed methods study. BMC Research Notes, 12**,** 1-6........
2. SEIFU, A., FANTAHUN, M. & WORKU, A. 2006. Reproductive health needs of out-of-school adolescents: a cross-sectional. Ethiopian Journal of Health Development, 20**,** 10-17.
3. IWELUNMOR, J., BLACKSTONE, S., NWAOZURU, U., CONSERVE, D., IWELUNMOR, P. & EHIRI, J. E. 2018. Sexual and reproductive health priorities of adolescent girls in Lagos, Nigeria: findings from free-listing interviews. International journal of adolescent medicine and health, 30.

**Not focused exclusively on young people (n=2)**

1. MASEMOLA-YENDE, J. 2015. Access to information and decision making on teenage pregnancy prevention by females in Tshwane. *Curationis,* 38**,** 1-9.
2. OSAGIEDE, E., TOBIN, E., ABAH, S., AWUNOR, N. & EHIMEN, F. 2016. Assessment of knowledge and sexual behaviour among undergraduates in a Nigerian tertiary institution. *Nigerian Journal of Medicine,* 25**,** 78-85.

**Focused on emergency contraception which is received after risky sexual intercourse (n=26)**

1. ABATE, M., ASSEFA, N. & ALEMAYEHU, T. 2014. Knowledge, attitude, practice, and determinants emergency contraceptive use among women seeking abortion services in Dire Dawa, Ethiopia. PloS one, 9**,** e110008.
2. ABRHA, S., ZERATSION, F., MOLLA, F., ETICHA, T., ASSEN, A. & MELKAM, W. 2014. Assessment of knowledge, attitude and practice among regular female preparatory school students towards emergency contraceptives in Mekelle, northern Ethiopia. International Journal of Pharma Sciences and Research (IJPSR), 5**,** 856-864.
3. AHMED, F. A., MOUSSA, K. M., PETTERSON, K. O. & ASAMOAH, B. O. 2012. Assessing knowledge, attitude, and practice of emergency contraception: a cross-sectional study among Ethiopian undergraduate female students. BMC public Health, 12**,** 1-9.
4. BUSERY, S. & SISAY, M. 2016. Knowledge, attitude and practice of emergency contraceptives among graduating female students of college of health and medical sciences, Haramaya University, Eastern Ethiopia. Sch Acad J Pharm, 5**,** 413-20.
5. FEKADU, Y. 2017. Knowledge attitude and utilization of emergency contraception among health science and medical students of Arba Minch University, 2015.
6. GEBREMEDHIN, K., GEBRESILLASSIE, T., BIHONE, B., DEMEKE, T. & HABTIE, N. 2017. Assessment of Knowledge, Attitude and Behaviour towards Emergency Contraceptive among Female Students of Fasiledes Preparatory School, Gondar, Ethiopia. Clin in Mother Child Health, 14.
7. GIRMA, T., EJETA, E., DECHASA, A. & ABDULKADIR, K. 2015. Knowledge, attitude and practices of emergency contraception among female students in preparatory school of East Shoa, Adama, Ethiopia. Gynecol Obstet (Sunnyvale), 5**,** 2161-0932.1000.
8. HAILEMARIAM, T. G., TESFAYE, T., MELESE, T., ALEMAYEHU, W., KENORE, Y., LELAMO, Y., SAUL, T. & SEIFU, C. N. 2015. Sexual experiences and emergency contraceptive use among female university students: a cross-sectional study at Wachamo University, Ethiopia. BMC research notes, 8**,** 1-8.
9. JIMA, A., SEGNI, M. T. & ZERGAW, A. 2016. Assessment of knowledge, attitude and utilization of emergency contraception among unmarried women of reproductive age in Adama, Ethiopia. Health Science Journal, 10**,** 0-0.
10. KEBEDE, A. 2009. Assessment of Knowledge, Attitude and Practice on Emergency contraception among Secondary, Preparatory, and Technical & Vocational School Female Students in Maichew Town, Southern Zone of Tigray, Ethiopia [PhD Thesis]. Addis Ababa University.
11. LENJISA, J. L., GETACHEW, Z. G., TOLA, N. L., KIFLE, S. T., GETACHEW, D., BEKELE, G. D. D. & WOLDU, M. A. 2013. Knowledge, attitude and practice of emergency contraceptives among ambo university female students, West Showa, Ethiopia. Research Journal of Pharmaceutical Sciences ISSN, 2319**,** 555X.
12. LENJISA, J., ULFINA, D., TAMME, E., KABA, G., BEREHE, H., LEMMA, N. & WOLDU, M. 2014. Knowledge and practice of emergency contraceptives among students at Ambo Techniques College, Ethiopia. Reprod Syst Sex Disord, 3**,** 2.
13. MAMUYE, S. A., WUDINEH, K. G., BELAY, A. N. & GIZACHEW, K. D. 2021. Assessment of Knowledge, Attitudes, and Practices Regarding Emergency-Contraception Methods among Female Dangila Hidase High School Students, Northwest Ethiopia, 2019. Open Access Journal of Contraception, 12**,** 1.
14. MEKURIA, M., TEFERI, E., HAILU, E. & JAIN, V. K. 2016. FACTORS ASSOCIATED WITH THE KNOWLEDGE, ATTITUDE AND PRACTICE OF EMERGENCY CONTRACEPTIVE AMONG METTU UNIVERSITY FEMALE STUDENTS, OROMIA REGIONAL STATE, WEST ETHIOPIA. Medico Research Chronicles, 3**,** 501-511.
15. MESFIN, D. 2020. Emergency contraceptive knowledge, utilization and associated factors among secondary school students in Wolkite town, southern Ethiopia, cross sectional study. Contraception and Reproductive Medicine, 5**,** 1-10.
16. MISHORE, K. M., WOLDEMARIAM, A. D. & HULUKA, S. A. 2019. Emergency contraceptives: knowledge and practice towards its use among Ethiopian female college graduating students. International journal of reproductive medicine, 2019.
17. NEGUSSIE, G. T., JIMA, A. & SHIFERAW, A. 2021. Assessment of Knowledge, Attitude and Utilization of Emergency Contraception Among Women of Reproductive Age in Arsi Zone, Ethiopia.
18. NIBABE, W. T. 2013. Female college students' knowledge, attitude and practice towards sex and emergency contraceptives.
19. SEIFU, M., GASHE, F., JEMAL, A., TESSEMA, S. & AMELO, W. 2016. Assessment of the knowledge, attitude and practice of EC and barriers to its use among the antenatal care seekers of Sululta Health Centers, Oromia region, Ethiopia. International Journal, 2**,** 29.
20. TAJURE, N. 2010. Knowledge, attitude and practice of emergency contraception among graduating female students of Jimma University, Southwest Ethiopia. Ethiopian journal of health sciences, 20.
21. TAMIRE, W. & ENQUESELASSIE, F. 2007. Knowledge, attitude, and practice on emergency contraceptives among female university students in Addis Ababa, Ethiopia. Ethiopian Journal of Health Development, 21**,** 111-116.
22. TEMESGEN, K., WORKIE, A. & TSEGAYE, D. 2017. Assessment of knowledge, attitude and practice towards emergency contraceptives and associated factors among Wollo University (Dessie Campus) undergraduate female students in Dessie, Ethiopia. J Epidemiol Public Health Rev, 2.
23. TILAHUN, E. 2010. Knowledge, Attitude and Practice on Emergency Contraception among Preparatory, and High School Female Students in Debrezeit Town Oromia Regional State, Ethiopia. Addis Ababa University.
24. WARRI, B. K. & GURMU, T. G. 2018. Knowledge, attitude and practice of progestin-only emergency contraceptives among female students of Jimma Teachers Training College, Jimma, Ethiopia. Ghana Medical Journal, 52**,** 183-188.
25. YEMANEH, Y., ABERA, T., HAILU, D., CHEWAKA, L. & NIGUSSIE, W. 2017. Knowledge, Attitude and Utilization towards Emergency Contraceptive among Preparatory Students of Mizan High School Students, Bench-Maji Zone, South West, Ethiopia, 2016. J Women's Health Care, 6**,** 2167-0420.1000400.
26. YITAYIH, G., YITAYIH, Y., SINTAYEHU, M., ASSEFA, Y. & ZEMENE, A. Evaluation of Awareness, Perception and Utilization Towards Emergency Contraceptive among Female Students in Private College at Bahir Dar, North West Ethiopia, 2014. emergency, 19**,** 20.
